# Cultural beliefs and health-seeking practices: Rural Zambians' views on maternal-newborn care

**DOI:** 10.1016/j.midw.2020.102686

**Published:** 2020-06

**Authors:** Julie M. Buser, Cheryl A. Moyer, Carol J Boyd, Davy Zulu, Alice Ngoma-Hazemba, Jessy Taona Mtenje, Andrew D. Jones, Jody R. Lori

**Affiliations:** a Department of Health Behavior and Biological Sciences, University of Michigan School of Nursing, 400 N. Ingalls, Ann Arbor, MI, 48109, United States; b Global REACH, University of Michigan Medical School, United States; c Departments of Learning Health Sciences and, Obstetrics & Gynecology, University of Michigan Medical School, United States; d Center for the Study of Drugs, Alcohol, Smoking & Health (DASH Center), University of Michigan, United States; e Women's Studies, LS&A, University of Michigan; f Institute for Research on Mothers & Gender, University of Michigan, United States; g Republic of Zambia Ministry of Health, Lundazi, Zambia; h School of Public Health, Department of Community and Family Medicine, University of Zambia, Lusaka, Zambia; i Africare-Zambia, Lusaka, Zambia; j Nutritional Sciences, Center for Human Growth and Development, School of Public Health, United States; k Department of Health Behavior and Biological Sciences, University of Michigan School of Nursing, United States

**Keywords:** Mewborn care, Maternal-newborn health, Cultural beliefs, Health-seeking practices, Rural Zambia, Maternal dualism

## Abstract

**Background:**

**-** Far too many newborns die or face serious morbidity in Zambia, as in many other sub-Saharan African countries. New knowledge is needed to enhance our understanding of newborn care and the cultural factors influencing the ways mothers seek newborn care. This study adds to the literature about rural Zambians’ cultural beliefs and practices related to newborn care and health-seeking practices that influence maternal-newborn health.

**Objective:**

**-** The goal of this study was to describe the factors associated with newborn care in rural Zambia.

**Design:**

**-** Sixty focus groups were conducted. Each group contained a minimum of 8 and maximum of 12 participants. Recruitment was conducted orally by word of mouth through the nurse in charge at the health facilities and village chiefs.

**Setting:**

**–** Data were collected between June and August 2016 in 20 communities located in Zambia's rural Lundazi (Eastern province), Mansa, and Chembe (Luapula province) Districts.

**Participants:**

**-** The study included community members (*n* = 208), health workers (*n* = 225), and mothers with infants younger than 1-year-old (*n* = 213).

**Findings:**

**-** The following themes emerged. From mothers with infants, the dominant theme concerned traditional and protective newborn rituals. From community members, the dominant theme was a strong sense of family and community to protect the newborn, and from health workers, the major theme was an avoidance of shame. A fourth theme, essential newborn care, was common among all groups.

**Key conclusions:**

**–** Together the themes pointed toward a maternal dualism for mothers in rural Zambia. Mothers with infants in rural Zambia likely experience a dualistic sense of responsibility to satisfy both cultural and health system expectations when caring for their newborns. Mothers are pulled to engage in traditional protective newborn care rituals while at the same time being pushed to attend ANC and deliver at the health facility. These findings can be used to understand how mothers care for their newborns to develop interventions aimed at improving maternal-child health outcomes.

**Implications for practice:**

**-** There were findings about the culture-specific prevention of cough, care of the umbilical cord, and early introduction of traditional porridge that carry implications for nursing practice. There is an obvious need to reinforce the importance of partner testing for STIs during routine ANC even though there is a desire to preserve dignity.

## Highlights

• Mothers caring for newborns have a maternal dualism between cultural and health system obligations.
• Traditional newborn protective rituals were identified to help nurses provide health education.
• Family and community expressed a strong need to protect the newborn using traditional belief systems.

## Introduction

In Zambia, the newborn mortality rate is 34 per 1,000 live births (UNICEF, 2017) and the infant mortality rate is 44 per 1,000 live births (UNICEF, 2018). To promote improved newborn health outcomes in rural Zambia, new knowledge is needed to enhance our understanding of newborn care and cultural factors influencing the ways mothers seek newborn care. Several studies from low- and middle-income countries (LMICs) show cultural beliefs strongly influence behavior during pregnancy, childbirth, and care-seeking (Lang-Baldé and Amerson, 2018; Lori and Boyle, 2011; Maimbolwa et al., 2003; Raman et al., 2016). Grandmothers and other family members play important roles in maternal-newborn health (Gupta et al., 2015; Moyer et al., 2014). Clearly understanding the beliefs and health-seeking practices of rural Zambians from a cultural perspective will support healthcare workers to provide acceptable care and interventions to improve maternal-child health.

The infant mortality in Eastern and Luapula Provinces are much higher than the overall Zambian national infant rate of 44 per 1,000 live births (Chief Statistics Office, 2015). Table 1 displays the demographic information for Eastern and Luapula Province (Chief Statistics Office, 2015).

**Table 1:** Demographics of Eastern and Luapula Province in 2015 (Chief Statistics Office, 2015).

Demographic	Eastern Province	Luapula Province
Population	1,813,445	1,127,453
Crude Birth Rate (CBR)	45.3 births per 1,000 population	47.2 births per 1,000 population
Crude Death Rate (CDR)	15.6 deaths per 1,000 population	16.9 deaths per 1,000 population
Infant Mortality Rate	94.9 per 1,000 live births	95.6 per 1,000 live births
Total Fertility Rate	6.2 births per woman	6.8 births per woman

Research in Zambia has focused on specific aspects of newborn care and health-seeking practices such as cultural care practices (Maimbolwa et al., 2003), umbilical cord care (Hamer et al., 2015; Herlihy et al., 2013; Sacks et al., 2015; Siwila, 2015), skin and thermal care (Sacks et al., 2015); antenatal care (ANC) seeking (Gabrysch et al., 2016), and postnatal care (PNC) (Sacks et al., 2017). Maimbolwa et al., (2003) explored cultural childbirth practices and beliefs in Zambia; however, there remains scant research documenting recent, broader knowledge and beliefs about newborn care in the country. Recognizing the limited research investigating cultural beliefs and health-seeking practices, the purpose of this focus group study was to determine the factors associated with newborn care in rural Zambia. This is important to influence best health practices in the future to improve newborn outcomes.

## Methods

### Design

We used a qualitative exploratory study design employing a semi-structured interview guide with 60 focus groups, with approximately eight participants in each group were conducted, and a semi-structured interview guided the data collection. Focus groups were conducted between June and August 2016 in 20 communities located in Zambia's rural Lundazi (Eastern province), Mansa, and Chembe (Luapula province) Districts. Research assistants served as interpreters. Focus groups were all conducted in the local languages of Tumbuka (Lundazi district) or Bemba (Mansa/Chembe districts). Few people in rural Zambia are able to read or write English. Tumbuka and Bemba are oral, not written, languages.

### Setting

The study sites were chosen because they were included as part of an earlier parent study conducted from 2015-2018 to determine the impact of maternity waiting homes (MWHs) on facility delivery among women living at least 10 km from health facilities in rural Zambia (Scott et al., 2018). The purpose of MWHs is to provide a setting where mothers can be accommodated during the final weeks of their pregnancy near a facility capable of providing essential emergency obstetric and newborn care (WHO, 1996). Parent study researchers conducted a mixed methods quasi-experimental impact evaluation of their MWH model using a controlled before-and-after design (Scott et al., 2018). There were total of 29 eligible health facilities that were located ≤2 h driving time to a referral facility and performed a minimum of 150 deliveries per year. Of those, 22 (76%) met one of the two sets of eligibility conditions outlined in detail in the protocol paper by Scott et al. (2018). Researchers worked with the local Ministry of Health to identify 10 intervention sites then selected comparison sites, matched to intervention sites on annual delivery volume and distance to a referral hospital (Scott et al., 2018). This focus group study compliments research included in the parent study but provides new information not presented elsewhere.

### Study population and sample

#### Inclusion criteria

A purposive sample included three different demographic groups in Lundazi and Mansa/Chembe Districts in Zambia: (1) Zambian mothers with infants younger than 1-year-old; (2) male and female community members older than 18 years, and (3) male and female professional and community health workers. These three groups were selected because they were believed to represent different perspectives and beliefs pertaining to health-seeking practices about maternal-newborn care. Health workers and community members were included in the focus group study recognizing their input undoubtedly influences maternal knowledge of newborn care and support for care-seeking behavior of women with infants younger than 1-year-old. Mothers with infants were invited to participate in the study even if they were younger than 18 years old. In Zambia, pregnant girls are considered “emancipated minors” if aged 15 years and older. There were 12 women between the ages of 15-17 included in the FGD for mothers. All literacy levels were eligible to participate. Only self-identified permanent residents of the study site villages were included. All other community members were excluded.

### Recruitment

Recruitment for the study was conducted by word of mouth through the nurse in charge at the health facilities and village chiefs. Village chiefs from ten communities in each district were informed in advance of when the nurse researcher would be coming to their area. The primary investigator supervised recruitment and data collection activities. The Zambian research assistant served as an interpreter and was fluent in both English and the local language. Participants who met eligibility requirements self-selected to participate on a first come basis and were turned away only when the maximum number of participants per group was reached. We arranged separate groups for mothers, community members, and health workers. Participants were provided a small snack and drink for their time in the focus group.

### Ethics consideration

Informed, verbal, and written consent were obtained from focus group participants before discussions. Institutional Review Board (IRB) approval was obtained before beginning the study from the University of X (HUM00110404), the Zambian IRB equivalent, Excellence in Research Ethics and Science (Ref. No. 2015-Dec-014), and the Zambian National Health Research Authority.

### Study instrument

The semi-structured focus group interview guide was developed from the maternal-newborn health literature (WHO & UNICEF, 2015). The focus group guide was pre-tested for conceptual and cultural meaning in the local contexts and minor changes were made accordingly. Table 2 displays selected interview questions and probes.

**Table 2:** Sample interview questions and probes.

Interview questions	Probes
Who do you go to for answers, guidance, and advice if you have a problem or question about pregnancy, childbirth or newborns?	How do they influence the decisions you make about your health and the health of your baby?
	How do you get the resources you need to change things that involve the health of your baby?
Tell me some things you or the midwife does right after a baby is born—in the first day.	Do new mothers or midwives do anything special to keep a baby warm right after it is born?
	In your communities, when does a newborn baby get the first bath? How often do you bathe a newborn?
How do you care for the newborn's umbilical cord?	Do you have the resources to care for the cord in the way you were taught?
	If you don't have the resources, what can you do?
Tell me how long a mother usually breastfeeds after her baby is born in your community.	How are mothers supported to breastfeed?
	When are other foods besides breast milk usually introduced?
Tell me about taking babies to the clinic for “routine” care.	Do mothers have their baby immunized? Why or why not?
	Does the newborn currently receive health care? If so, where do they receive health care?
Tell me about taking babies to the clinic for “sick’ care.	Tell me what you do if the baby has a cough. What causes the cough?
	Tell me what you do if the baby has diarrhea. What causes diarrhea?
What are some things mothers can do to keep their baby healthy?	Do you have the resources to do these things?
	Is there any problem to do these things?

### Data collection

The study included rural Zambians *(n* = 646), comprised of groups of community members (*n* = 208), health workers (*n* = 225), and mothers with infants younger than 1-year-old (*n* = 213). The groups of health workers included both professional and community health workers. Professional health workers (i.e. nurses, midwives) receive formal training while lay community health workers are locally trained as part of a national safe motherhood program through the Zambian Ministry of Health to teach pregnant women about the importance of delivering in a facility, having a birth plan and practicing healthy behaviors during pregnancy and early childhood (Saving Mothers, Giving Life, 2018). The groups of community members and health workers were heterogeneous with varying ages, compositions of males and females, number of living children, educational level, and length of time in the community. The groups of mothers with infants less than one year of age were more homogeneous.

Each group contained a minimum of 8 and maximum of 12 participants. The interviews lasted approximately 60 minutes. A semi-structured interview guide was used to explore how rural Zambians understand and describe newborn care and health-seeking. Interviews were digitally audiotaped, transcribed verbatim, and 20% were back-translated. No names were used in the focus groups or on the audiotapes. Interpreters and group members were asked to avoid discussing group content outside of the group.

### Data analysis

An inductive iterative process was employed to analyze the focus group data using the four main stages identified by Bengtsson (2016): decontextualization, recontextualization, categorization*,* and compilation. Through decontextualization, the transcriptions were read and reread to obtain a sense of the whole. Data were coded and evaluated for significance. Coding was deductive, using a detailed codebook, and inductive, allowing for themes to emerge from the data.

The original text was then reread alongside the final list of meaning units through a process of recontextualization (Bengtsson, 2016). Categorization with latent content analysis was used to identify themes and categories. The primary investigator sorted and classified by similar thematic content and separated into smaller categories based on the aims of the study. Finally, meanings in the text were identified and compiled to present as quotes.

## Findings

Demographic characteristics of the focus group study participants are shown in Table 3. Overall, most participants were women (71.6%) and married (86.8%). The average age was 37 years, while the average time living in the community was 25 years. Among the 646 total participants, focus groups were made up of a similar number of participants among community members (*n* = 213), health workers (*n* = 208), and mothers with infants younger than 1-year-old (*n* = 225). In terms of educational level, 6.2% had no formal education, 12.5% had a lower primary level (Grades 1-4), and 32.7% had an upper primary level (Grades 5-7) of education.

**Table 3:** Focus group participant characteristics.

Demographic Characteristic	Total	Mothers w/ Infants <1yr	Community Members	Health Workers
	(*n* = 646)	33.0% (*n* = 213)	32.2% (*n* = 208)	34.8% (*n* = 225)
District	% (*n*)	% (*n*)	% (*n*)	% (*n*)
Lundazi	49.2% (318)	49.3% (105)	51.4% (107)	47.1% (106)
Mansa/Chembe	50.8% (328)	50.7% (108)	48.5% (101)	65.1% (119)
**Age (years)**				
Range	15-88	15-65	18-77	18-88
Mean (SD)	37 (13.3)	26 (8)	39.3 (11.9)	45 (11.5)
Missing	0.5% (3)	0.5% (1)	0.5% (1)	0.4% (1)
**Sex**				
Male	28.4% (183)	None	38.0% (79)	44.0% (99)
Female	71.5% (462)	100% (213)	62.0% (129)	55.6% (125)
Missing	0.2% (1)	None	none	0.4% (1)
**Marital status**				
Married	85.9% (555)	89.2% (190)	88.0% (183)	80.9% (182)
Single	4.6% (30)	1.9% (4)	4.3% (9)	7.6% (17)
Widowed	2.6% (17)	1.4% (3)	2.9% (6)	3.6% (8)
Separated/Divorced	5.9% (38)	6.6% (14)	4.3% (9)	6.7% (15)
Missing	0.9% (6)	0.9% (2)	0.5% (1)	1.3% (3)
**Number of living children**				
0	2.2% (14)	0.5% (1)	4.3% (9)	1.8% (4)
1-5	62.7% (405)	84.0% (179)	58.2% (121)	46.7% (105)
6 and above	34.5% (222)	15.0% (32)	36.5% (76)	50.7% (114)
Range	0-16	0-9	0-16	0-14
Mean (SD)	4.5 (2.7)	3.2 (2.0)	4.7 (2.7)	5.5 (2.7)
Missing	0.8% (5)	0.5% (1)	1.0% (2)	0.9% (2)
**Education level**				
None	4.8% (31)	7.0% (15)	4.8% (10)	2.7% (6)
Lower (1-4) & Upper Primary (5-7)	42.4% (274)	49.3% (105)	43.8% (91)	34.7% (78)
Junior (8-9) & Senior Secondary (10-12)	49.2% (318)	39.4% (84)	49.5% (103)	58.2% (131)
Tertiary	2.2% (14)	0.9% (2)	1.4% (3)	4.0% (9)
Missing	1.4% (9)	3.3% (7)	0.5% (1)	0.4% (1)
**Time in community (years)**				
Range	1-88	1-65	1-68	1-88
Mean (SD)	25.2 (16.1)	16.8 (11.3)	26.9 (15)	31.6 (17.5)
Missing	0.5% (3)	0.5% (1)	0.9% (2)	none

### Themes

The following themes were identified from each of the groups independently: from mothers with infants younger than 1-year-old, (1) traditional newborn protective rituals; from community members, (2) a strong sense of family and community to protect the newborn, and from health workers, (3) an avoidance of shame. A fourth theme, essential newborn care, was common among all groups. Table 4 summarizes themes and categories from the focus groups.

**Table 4:** Summary of themes and categories emerging from focus groups.

Group	Theme	Category
Mothers with infants under one year	Traditional newborn protective rituals	Prevention of cough and pneumonia
		Care of the umbilical cord
		Early introduction of porridge
Community members	Strong sense of family & community to protect the newborn	Husbands and maternal-newborn health
		Grandmothers and maternal-newborn health
		Community members and maternal-newborn health
Health workers	Preservation of dignity	Cultural concerns related to maintaining privacy
		Social concerns about partner's fear of HIV/STI testing.
Mothers with infants under one year, community members, & health workers	Essential newborn care	Pregnancy and postpartum care
		Breastfeeding
		Newborn danger signs
		Immunizations

### Mothers with infants younger than 1-year-old

#### Traditional newborn protective rituals

Participants most often mentioned using traditional newborn protective rituals when caring for newborns. Categories of protective rituals included prevention of cough and pneumonia, care of the umbilical cord, and early introduction of porridge to the newborn.

##### Prevention of cough and pneumonia

Mothers with infants described the ritual use of fire and sperm to prevent cough and pneumonia. The belief, as explained by interpreters in both districts, is the sperm of the man will make the baby strong and prevent cough. The words used to label traditional practices were different based on whether participants spoke Bemba or Tumbuka, but the essence of the health belief was similar in both districts. As one mother with an infant younger than 1-year-old in Lundazi District explained:

When the baby is a month old we prepare fire in the house where we live. Then we will have sex that night [and] after having sex we will spread the sperms to the joints of the baby. After this [is] done, we pass the baby back and forth over the fire to make the baby strong and keep from unnecessary coughs.

In both districts, ritual of preparing a special fire and protective use of sperm is usually performed when the baby is about 1 month old. Traditional herbs are placed in the fire with the expectation that the smoke will clear the lungs and avert cough. As a woman with a baby younger than 1-year-old in Mansa District described:

When a child has a cough it means the father to the child was having sex with other mothers and when he returned home he touched the child. They would be advised by elders to have sex then [the] husband must release his sperms on his hand to spread [over] the sick baby.

Mothers with infants in the focus group study acknowledged they usually do not talk about traditional newborn protective rituals involving the prevention of cough and pneumonia at the rural clinics because they've been told by health workers not to engage in cultural newborn care.

##### Care of the umbilical cord

Many mothers with infants mentioned the use of herbs and powders on the umbilical cord to make it heal and fall off faster so they can carry the baby on their back. Grandmothers, in particular, promote the practice of applying traditional herbs to the umbilical cord. A mother with a baby younger than 1-year-old from Lundazi explained:

With my other children, I used traditional herbs which my grandmother had brought from the villages. I heard from friends that women often use traditional herbs like from the flower of pumpkin pumpkin plants or even others use rate feces on the cord in hopes it will heal and fall off faster.

##### Early introduction of traditional porridge

In both districts, early introduction of traditional herbs mixed as a porridge when the child is about 1 month old was reported to “make the baby strong and healthy.” According to a mother in Mansa District, “We give herbs mixed with porridge at 1 month to keep them from getting diseases.” However, mothers in the study with infants younger than 1-year-old mentioned they do not discuss the use of herbs at the clinic because they would be “scolded by midwives.”

### Community members

#### Strong sense of family and community to protect the newborn

Among focus groups with community members, the main theme was a *strong sense of family and community to protect the newborn*. Focus groups in both geographical areas with community members expressed a strong desire to protect newborns and play important roles in maternal-newborn health.

##### Husbands and maternal-newborn health

Participants in Lundazi and Mansa/Chembe Districts focus groups talked about the importance of “following the wishes” of the husband when deciding how to protect the newborn and when to go to the clinic. As described by a female community member in Mansa District:

For us, it is the husband and the mother of our husband who makes sure we follow some of the customs and traditions. Usually if a woman is pregnant then the husband will choose where to deliver. It is for the husband to decide if the woman can go to the clinic with the baby for check-ups.

##### Grandmothers and maternal-newborn health

Grandmothers were described as a possessing a strong sense of responsibility to protect the newborn and greatly influencing a woman's decision to follow cultural or health system newborn care practices in rural Zambia. Several community members in both districts described the role of grandmothers and the use of traditional medicine in maternal-newborn health. A female community member in Lundazi stated:

When we have prolonged labor in the village, our grandmothers prepare medicine to ease the pain and deliver faster. This is known and it's not allowed in the hospital because they say it may destroy both the life of the child and mother.

Traditional herbs are often used in cultural maternal-newborn care practices in rural Zambia, but mothers are reluctant to divulge their use because health workers do not allow them to be used in facilities. In the health system, nurses and midwives spread health education messages about the potentially harmful effects of traditional medicine used in maternal-newborn care. Grandmothers assist mothers who are interested in speeding labor to avoid pain. Community members expressed the need to follow the advice of the “elder mothers” because “they know what is best for mothers.” Nurses and midwives stressed the need for health education targeted towards grandmothers. One midwife in Mansa commented:

During labor at home, the grandmother and maybe other people like the neighbors, there used to be a lot of people in the room when the mother was delivering. They forced the mother to push or maybe tie a chitenge [traditional fabric] material around the waist of the mother then pulled it so that she gives birth quickly. But all that stopped after educating the mothers-in-law now they just come to the clinic. Other people still they believe in tradition herbs which they use when they are at home, therefore my appeal to midwives I work with is that we should continue educating grandmothers on the importance of delivering at the facility.

##### Community members and maternal-newborn health

Along with family, community members in rural Zambia have a strong sense of responsibility to protect newborns. In focus groups with community members, there was an often-cited belief that no single or unmarried people in the community should touch the newborn baby for at least one month or the baby risks death or infertility. According to a female community member in Mansa District:

Immediately [after] a baby is born only selected people are to touch the baby like the grandmothers and other elderly people but not and strictly not the singles or divorces for fear that the child may die in case they are just from having sex which is considered to be dirty to touch the baby.

After one month, mothers rely on single female family members to provide support in caring for the infant. Community members in the study spoke about how neighbors and friends of mothers have a “duty to protect” everyone in the village “no matter what their age.”

##### Social concerns about HIV/STI testing

During the first ANC visit, per Zambian Ministry of Health officials, guidelines stress the importance of partner testing for HIV and various STIs. A female health worker in Mansa explained, “Men don't like to be tested with women for HIV/STIs.” A male health worker in Mansa said, “Three-quarters of our men do not come for tests like HIV, syphilis and other diseases [because] they say it is for women only. Hence, they end up delivering from home.” In Lundazi, a health worker stated:

The first antenatal visit is a challenge to most of the pregnant mothers because husbands refuse to go with their wives for fear of being tested for HIV/AIDS and syphilis. Husbands say once they know their status is positive he will kill himself to avoid being known by family members, friends, and community that he is sick.

Health workers in the study said that the most common reason men do not participate in antenatal pregnancy care is their desire to avoid STI testing.

### Health workers

#### Preservation of dignity

The theme from focus groups with health workers was the cultural and social *preservation of dignity*. In both districts, when health workers were asked to discuss reasons for mothers not seeking ANC, participants mentioned: lack of privacy at the clinic and partner's fear of HIV/STI (sexually transmitted infections) testing.

##### Cultural concerns related to maintaining privacy

Health workers frequently said a desire by mothers not to be seen naked by male nurses in the maternity ward led to hesitancy to deliver at the facility. According to a female health worker in Lundazi, “Mothers don't attend ANC because they feel shy and fear exposure to opposite sex health personnel at the clinic.” Along this line was the frequent discussion of a lack of privacy in the tight quarters of a delivery room. A female health worker in Mansa explained:

There's no privacy at the facility because they are using a [converted] office as an examination place. [This] makes the woman uncomfortable to deliver at the facility because there is no maternity ward. If the midwife wants to talk in a labor ward everyone outside hears.

There is a widely held belief in rural Zambia that no man outside of the home should see a woman without clothes on. Furthermore, pregnant women believe it is embarrassing if others hear them groan or cry during labor. None of the participants mentioned sexual harassment by male nurses.

### Common to all focus groups

#### Essential newborn care

The common theme across all types of focus groups was an understanding of *essential newborn care*. Responses were placed in the theme of *essential newborn care* if focus group participants mentioned newborn care and care-seeking according to pregnancy, childbirth, postpartum, and newborn care (PCPNC) guidelines by the WHO and UNICEF (2015). The PCPNC includes recommendations from approved WHO guidelines relevant to maternal and perinatal health including: preeclampsia and eclampsia, postpartum hemorrhage, postnatal care for the mother and baby, newborn resuscitation, prevention of mother-to-child transmission of HIV, HIV and infant feeding, malaria in pregnancy, interventions to improve preterm birth outcomes, tobacco use and secondhand exposure in pregnancy, postpartum depression, postpartum family planning, and post-abortion care (WHO & UNICEF, 2015). The categories supporting this theme include *pregnancy and postpartum care, breastfeeding, newborn danger signs, and immunizations.*

##### Pregnancy and postpartum care

Many participants in all groups were able to state the importance of *pregnancy and postpartum care* including attending ANC, facility delivery, and PNC, and correctly identified maternal-newborn danger signs as outlined in the PCPNC. As one mother with a baby younger than 1-year-old in Mansa mentioned:

Family and friends advise us to go early for antenatal visits because ladies don't hide any secrets. They also tell us to go for medications in case the baby is not in a good position to help your friend from dying. Sometimes if a person is pregnant she is in danger and should go to the clinic.

In Lundazi, a female community member commented that after birth in the clinic, “They discharge mothers and tell her to come at 6 days for postnatal checkups for both the baby and the mother and also third postnatal at 6 weeks to check for the baby.”

#### Breastfeeding

The majority of participants in all groups in both districts were able to identify the importance of *breastfeeding*. A mother with a baby younger than 1-year-old commented, “At the clinic they emphasize the mother to start breastfeeding the baby immediately after the baby is born and they discharge the mother only after first seeing the baby has started breastfeeding.” A male health worker in Mansa mentioned, “The mother should breastfeed the child the first milk because it is the most nutritious milk ever. The mother should be clean always and the baby too.”

##### Newborn danger signs

Most focus group participants in both districts correctly cited *newborn danger signs* as reasons for taking newborns to the clinic, such as convulsions, difficulty breathing, and fever. A female community member in Lundazi District noted, “These days we take the baby to the clinic either coughs, fever or fitting [seizures].” A male community member in Mansa said the community health workers, “Emphasize to us to bring the baby to clinic to see if the baby is gaining or losing because some gain more weight abnormally because some do reach that line when they have feeding difficulties.”

##### Immunizations

In all groups, most participants expressed understanding of the importance of returning to the clinic for *immunizations* as the newborn grows. As stated by a female community member in Mansa, “We need to observe hygiene and the food which the baby may eat should be soft and also the vaccines like polio, BCGs and all the 4 injections rotavirus and PCV [pneumococcal conjugate vaccine] are necessary.”

### Similarities and differences among focus groups

Mothers with infants younger than 1-year-old, community members, and health workers in both districts mentioned the same theme pertaining to *essential newborn care.* Themes identified among communities with and without a MWH were similar. In all communities, mothers mentioned feeling pulled to maintain traditional pregnancy care while recognizing the benefits of receiving care at a health facility. Mothers attempt to straddle this divide by hiding the use of traditional herbs while accessing care at a facility. A mother in Lundazi district commented:

Because of our ancestral customs and norms we have fear of disobeying our grandmothers and husbands to go to the facility for delivery even if others advise us to start using the facility. We fear to die due to [long] distances from help and want to go to the facility but our traditional rules from elders in the communities, like grandmothers, threaten pregnant mothers saying if you deliver at the hospital you will not manage [and] the doctor will not help you. They say home delivery is better because you will be given lupusu (traditional herbs) to help you deliver faster. Hence, to appease the elders, we take these herbs with us to the facility but we have to hide them from the nurses because they will throw them away and scold us for using them.

**Fig. 1:**
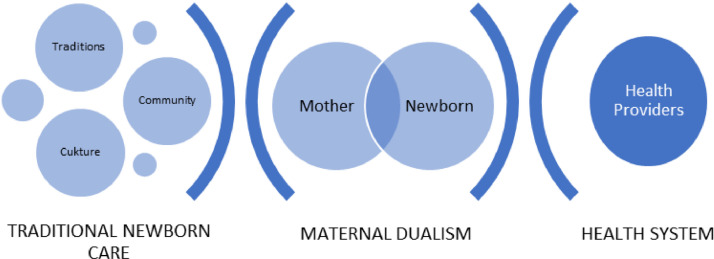
Maternal Dualism between traditional newborn care and health system in rural Zambia.

Fig. 1 illustrates the maternal dualism experienced by mothers. The similarity in themes brought up independently by focus groups across geographic areas was surprising. We expected there would be distinct differences in responses between districts where focus groups were conducted. We anticipated finding more differences given the likely range between cultures, education, stages of development, population diversity, and access to health care between districts. Findings point to wide-ranging pervasive cultural and health system influences on newborn care in rural Zambia.

**Fig. 2:**
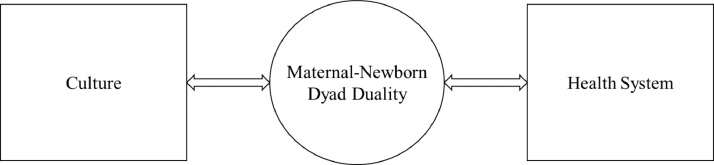
Maternal Dualism between traditional newborn care and health system in rural Zambia.

Community members were least likely to mention the essential newborn care theme compared to mothers with infants and health workers in both districts. On the whole, health workers were more expressive, giving a greater number of total responses with longer replies to questions than mothers with infants and community members. Regarding the understanding of essential newborn care, it is predictable health workers mentioned the theme a greater number of times than other groups. This could be explained by higher levels of education and literacy leading to a greater knowledge of maternal-newborn health care among health workers.

## Discussion

Our qualitative exploratory study explored newborn care beliefs and health-seeking practices while examining the cultural and health system factors associated with the ways women seek care from the point of view of rural Zambian mothers with infants younger than 1-year-old, community members, and health workers. This study adds to the literature about cultural beliefs and practices of rural Zambians related to newborn care and health-seeking practices influencing maternal-newborn health.

### Substantiation of themes

Findings from focus groups relating to *traditional newborn protective rituals* (macrosystem) are consistent with various other studies in Zambia. Participants relayed culturally specific prevention of cough, care of the umbilical cord, early introduction of traditional porridge, familial roles, and community support. The use of traditional herbs and powders when caring for the umbilical cord corroborates findings from other studies in Zambia (Hamer et al., 2015; Herlihy et al., 2013; Maimbolwa et al., 2003; Sacks et al., 2015; Siwila, 2015).

Participants were knowledgeable about *essential newborn care*. Overall, knowledge among participants revealed an understanding about the importance of exclusive breastfeeding while results related to the early initiation of complementary foods as protective factors is similar to findings reported by Gewa and Chepkemboi (2016) and Katepa-Bwalya et al. (2015). The importance of familial roles and involvement of family members in mother-newborn care seeking in Zambia has been documented previously (Gabrysch et al., 2016; Sialubanje et al., 2015, 2016) as has the use of traditional medicine during labor (Dika, Dismas, Iddi, & Rumanyika, 2017; M'soka, et al., 2015). While it is commendable that community members mentioned an understanding of newborn care in focus groups, their discussion of this theme being less frequent than other groups focuses our attention on the importance of targeting community members for maternal-newborn health education.

Preservation of dignity, reasons for not seeking antenatal care, and motives for home delivery were also identified by other studies in Zambia (Lori, et al., 2016; Phiri et al., 2014; Sacks et al., 2017; Sinyange et al., 2016). The desire to preserve dignity and avoid being shamed by lack of privacy at the clinic and fear of receiving partner testing for STIs are important findings for health care providers to keep in mind when caring for pregnant women. To preserve dignity, changes are needed in rural Zambia to ensure the environment and facilities are mother and baby friendly (International Confederation of Midwives, White Ribbon Alliance, International Pediatric Association, & World Health Organization, 2015).

### Maternal dualism

Linked together, the themes emerging from focus groups point toward a maternal dualism for mothers in rural Zambia. Mothers with infants likely experience a dualistic sense of responsibility to satisfy both cultural and health system expectations when caring for their newborns (Fig. 1). Mothers are pulled to engage in traditional protective newborn care rituals while at the same time being pushed to attend ANC and deliver at the health facility. Contributing to this dualism felt by mothers many mothers face fines by chiefs and community leaders for failing to attend ANC or deliver at the health facility. In Zambia, Greeson and colleagues (2016) researched the frequency and perception of penalties for home delivery and found while communities largely supported the use of penalties to promote facility delivery, the penalties introduced a new tax on poor rural mothers and may have deterred their utilization of postnatal and child health care services. Researchers viewed the imposition of penalties as a punitive adaptation can impose new financial burdens on vulnerable mothers and contribute to widening health, economic, and gender inequities in communities (Greeson et al., 2016). These penalties add an additional layer of complexity and probable pressure on mothers to meet health system responsibilities.

The emergence of the *strong sense of family and community to protect the newborn* theme in focus groups with community members adds strength to the argument that mothers are pulled to meet cultural responsibilities placed on them by those outside the maternal-newborn dyad. Mothers likely feel pressured by husbands, grandmothers, and community members to maintain rural Zambian cultural beliefs about newborn care. In the health workers’ focus groups, the uncovering of the preservation of dignity theme sheds light on the dichotomy mothers feel when considering access to the rural Zambian health system. According to health workers, mothers face barriers to meeting health system responsibilities because of a lack of privacy in facilities and a reluctance by their husbands to undergo partner testing for STIs. Again, even in the minds of health workers, mothers are being pulled in two different directions. They might recognize the need to meet health system responsibilities, but they feel compelled to satisfy responsibility to culture and family.

The maternal dualism felt by mothers to fulfill both cultural and health system responsibilities when caring for their newborns undoubtedly causes undue stress and anxiety for new mothers. Future studies should explore this maternal dualism to better understand how nurses and midwives can meet the psychosocial needs of this population. In rural Zambia, individual interviews with mothers rather than focus groups could be considered for a follow-up study allowing for confidentiality to be fostered as participants might be reluctant to express maternal dualism in the open forum of a focus group.

### Implications for practice

Numerous implications for nursing practice emerged from this investigation. Traditional cultural practices reflect values and beliefs held by members of a community for periods often spanning generations (OHCHR, 1995). Nurses and midwives can promote the maintenance of cultural beliefs that benefit or at the very least do no harm to the mother-newborn dyad while encouraging the reframing of potentially detrimental practices.

There were findings about the culture-specific prevention of cough, care of the umbilical cord, and early introduction of traditional porridge that carry implications for nursing practice. For example, the traditional protective rituals to prevent cough and pneumonia involving fire and sperm have long-standing cultural roots and could be maintained – provided the newborn is not exposed to smoke for extended periods of time and the risk of burns is mitigated. On the other hand, the application of potentially harmful herbs and powders on the umbilical cord should be discouraged.

To address the reluctance of rural Zambians to place newborns on their back to sleep until the cord falls off, nurses and midwifes can deliver culturally appropriate messages about safe sleep. Health professionals should educate mothers that when the newborn is not placed in a supine position to sleep it may contribute to aspiration or choking (NICHD, 2018) or place the child at risk for Sudden Infant Death Syndrome (AAP, 2016). Meanwhile, the introduction of traditional herbs mixed as a porridge at 1 month of age should be discouraged. The WHO (2018b) recommends infants start receiving complementary foods at 6 months of age in addition to breast milk.

Regarding familial roles, nurses and midwives have a duty to foster those promoting the health of the mother-newborn dyad. Beliefs inspiring monogamy by partners during pregnancy should not be discouraged–especially in light of the dangers of mother-to-child transmission of HIV/AIDS. The belief that no single or unmarried person in the community should touch the newborn baby is a protective ritual practiced by community members reducing exposure to infection and controlling the spread of disease. The often-mentioned recommendation by grandmothers to use traditional medicines to speed labor should be approached with sensitivity by health care professionals. Many countries have their own traditional or indigenous forms of healing firmly rooted in their culture and history (WHO, 2013). Nurses, midwives, and community health workers can incorporate the potential for harmful effects to the mother and newborn into health education messages.

### Limitations

Several study design limitations are worth mentioning. We used a purposive sample in two rural districts in Zambia and results cannot be generalized nor do they reflect changes over time. Findings expose the experience of focus group participants recruited at rural primary care health centers. The viewpoints of those not accessing the Ministry of Health facilities were not obtained, therefore, findings might not be applicable to those not seeking care in the clinics. In addition, given language differences, it is possible that there were subtleties lost in translation between focus groups and the translation into transcripts. However, this was mitigated through back-translation of 20% of English-language transcripts to ensure that the meaning was properly interpreted

## Conclusion

These findings shed light on the beliefs and practices of rural Zambian mothers, community members, and health workers related to newborn care and health-seeking practices. Traditional newborn protective rituals and professional newborn care practices were identified. Findings also revealed a strong sense of family and community to protect the newborn using traditional belief systems. Positively, rural Zambians have a general understanding of essential newborn care according to WHO guidelines. The study uncovered a maternal dualism between cultural and health system responsibilities faced by mothers caring for newborns as they strive to balance responsibilities associated with traditional protective newborn care rituals and essential newborn care practices. This focus group study lays the groundwork for developing future research to explore the push-pull felt by mothers navigating cultural practices and health system regulations to inform future interventions aimed at improving newborn care in rural Zambia where far too many newborns still face serious morbidity or death.

Moreover, a targeted exploration of the family's sense to protect the newborn is warranted to understand whether it is necessary to recommend policies to increase involvement by Zambian husbands and grandmothers in routine professional maternal-newborn health care. Additional research should investigate the roles of husbands, grandmothers, and community members and explore their understanding of the benefits of their involvement in pregnancy and postpartum maternal-newborn care given that newborn morbidity and mortality remain a serious global health challenge with almost 1 million newborns dying in the first day of life (UNICEF, 2016) in LMICs,

In conclusion, this study described knowledge and beliefs about newborn care while examining the social and cultural factors associated with the ways women seek care from the perspective of rural Zambian mothers, community members, and health workers. Similarities and differences in knowledge and beliefs of newborn care were explored to identify traditional and professional newborn care practices in rural Zambia. Findings can be used to inform future interventions aimed at improving maternal-newborn care.

## Author Credit Statement

**Julie M. Buser, PhD, CPNP-PC, RN, BA, corresponding author,**Primary author, conceived the original study and developed the protocol, drafting the article, analysis and interpretation of data, Substantial contributions to conception and design of analysis, Review and approval of final draft.

**Cheryl A. Moyer, PhD, MPH,** Conceived the original study and developed the protocol, Knowledge and ownership of the data, analysis and interpretation of the data, content expertise, Revising article critically for important intellectual content, Review and approval of final draft.

**Carol J Boyd, PhD, RN, FIAAN, FAAN,** Conceived the original study and developed the protocol, Knowledge of analysis and interpretation of the data, content expertise, Revising article critically for important intellectual content, Review and approval of final draft.

**Davy Zulu, MD,** Knowledge of the data, analysis, content expertise, Revising article critically for important intellectual content, Review and approval of final draft.

**Dr. Alice Ngoma-Hazemba, PhD, MPH, BSc.,** Knowledge of the data, analysis, content expertise, Revising article critically for important intellectual content, Review and approval of final draft.

**Jessy Taona Mtenje,** Knowledge of the data, analysis, content expertise, Revising article critically for important intellectual content, Review and approval of final draft.

**Andrew D. Jones, PhD,** Knowledge of the data, analysis, content expertise, Revising article critically for important intellectual content, Review and approval of final draft.

**Jody R. Lori, PhD, CNM, FACNM, FAAN,** Conceived the original study and developed the protocol, Knowledge and ownership of the data, analysis and interpretation of the data, content expertise, Revising article critically for important intellectual content, Review and approval of final draft.

## Ethical Approval

Institutional Review Board (IRB) approval was obtained before beginning the study from the University of Michigan Health Sciences and Behavioral Sciences Institutional Review Board (HUM00110404), the Zambian IRB equivalent, Excellence in Research Ethics and Science, and the Zambian National Health Research Authority.

## Funding Sources

We acknowledge the following sources of funding for the current study: the University of Michigan International Institute, 10.13039/100009999African Studies Center, Department of Afroamerican and African Studies and the South African Initiatives Office. The parent study program was developed and is being implemented in collaboration with Merck for Mothers, Merck's 10-year, $500 million initiative to help create a world where no woman dies giving life. Merck for Mothers is known as MSD for Mothers outside the United States and Canada (MRK 1846-06500.COL). The development of the parent study article was additionally supported in part by the Bill & Melinda Gates Foundation (OPP1130334) https://www.gatesfoundation.org/How-We-Work/Quick-Links/Grants-Database/Grants/2015/07/OPP1130334 and The ELMA Foundation (ELMA-15-F0010) http://www.elmaphilanthropies.org/the-elma-foundation/. The funders had no role in study design, data collection and analysis, decision to publish, or preparation of the manuscript. The content is solely the responsibility of the authors and does not necessarily reflect positions or policies of Merck, the Bill & Melinda Gates Foundation, or The ELMA Foundation.

## Declaration of Competing Interest

None to declare

## Appendix Supplementary materials

Image, application 1

## References

[bib0001] AAP, 2016. American Academy of Pediatrics. American Academy of Pediatrics Announces New Safe Sleep Recommendations to Protect Against SIDS, Sleep-Related Infant Deaths. Retrieved from: https://www.aap.org/en-us/about-the-aap/aap-press-room/pages/american-academy-of-pediatrics-announces-new-safe-sleep-recommendations-to-protect-against-sids.aspx.

[bib0006] Bengtsson M. (2016). How to plan and perform a qualitative study using content analysis. Nursing Plus Open.

[bib0007] Chief Statistics Office, 2015. Zambia demographics at a glance. Retrieved fromhttp://zambia.opendataforafrica.org/apps/atlas.

[bib0011] Gabrysch S., McMahon S.A., Siling K., Kenward M.G., Campbell O.M. (2016). Autonomy dimensions and care seeking for delivery in Zambia; the prevailing importance of cluster-level measurement. Sci. Rep..

[bib0012] Gewa C.A., Chepkemboi J. (2016). Maternal knowledge, outcome expectancies and normative beliefs as determinants of cessation of exclusive breastfeeding: a cross- sectional study in rural Kenya. BMC Public Health.

[bib0013] Greeson D., Sacks E., Masvawure T.B., Austin-Evelyn K., Kruk M.E., Macwan'gi M., Grépin K.A. (2016). Local adaptations to a global health initiative: penalties for home births in Zambia. Health Policy Plan..

[bib0014] Gupta M.L., Aborigo R.A., Adongo P.B., Rominski S., Hodgson A., Engmann C.M., Moyer C.A. (2015). Grandmothers as gatekeepers? The role of grandmothers in influencing health-seeking for mothers and newborns in rural northern Ghana. Global Public Health.

[bib0015] Hamer D.H., Herlihy J.M., Musokotwane K., Banda B., Mpamba C., Mwangelwa B., Grogan C. (2015). Engagement of the community, traditional leaders, and public health system in the design and implementation of a large community-based, cluster- randomized trial of umbilical cord care in Zambia. Am. J. Trop. Med. Hyg..

[bib0017] Herlihy J.M., Shaikh A., Mazimba A., Gagne N., Grogan C., Mpamba C., Messersmith L. (2013). Local perceptions, cultural beliefs and practices that shape umbilical cord care: a qualitative study in Southern Province, Zambia. PLoS One.

[bib0018] International Confederation of Midwives, White Ribbon Alliance, International Pediatric Association, & World Health Organization (2015). Mother− baby friendly birthing facilities. Int. J. Gynecol. Obstet..

[bib0021] Katepa-Bwalya M., Mukonka V., Kankasa C., Masaninga F., Babaniyi O., Siziya S. (2015). Infants and young children feeding practices and nutritional status in two districts of Zambia. Int. Breastfeed. J..

[bib0022] Lang-Baldé R., Amerson R. (2018). Culture and birth outcomes in sub-saharan Africa: a review of literature. J. Transcult. Nurs..

[bib0024] Lori J.R., Boyle J.S. (2011). Cultural childbirth practices, beliefs, and traditions in postconflict Liberia. Health Care Women Int..

[bib0027] Lori J.R., Munro-Kramer M.L., Mdluli E.A., Musonda G.K., Boyd C.J. (2016). Developing a community driven sustainable model of maternity waiting homes for rural Zambia. Midwifery.

[bib0029] Maimbolwa M.C., Yamba B., Diwan V., Ransjö-Arvidson A. (2003). Cultural childbirth practices and beliefs in Zambia. J. Adv. Nurs..

[bib0030] Moyer C.A., Adongo P.B., Aborigo R.A., Hodgson A., Engmann C.M., DeVries R. (2014). “It's up to the woman's people”: how social factors influence facility-based delivery in Rural Northern Ghana. Matern. Child Health J..

[bib0032] M'soka N.C., Mabuza L.H., Pretorius D. (2015). Cultural and health beliefs of pregnant women in Zambia regarding pregnancy and child birth. Curationis.

[bib0033] NICHD, 2018. National institute for child health and development safe to sleep campaign, baby's anatomy when on the stomach and on the back. Retrieved fromhttps://safetosleep.nichd.nih.gov/resources/providers/downloadable/baby_anatomy_image.

[bib0034] OHCHR, 1995. Fact sheet no.23, harmful traditional practices affecting the health of women and children. Retrieved fromhttps://www.ohchr.org/Documents/Publications/FactSheet23en.pdf.

[bib0035] Phiri S.N.A., Fylkesnes K., Ruano A.L., Moland K.M. (2014). ‘Born before arrival’: user and provider perspectives on health facility childbirths in Kapiri Mposhi District, Zambia. BMC Pregnancy Childbirth.

[bib0037] Raman S., Nicholls R., Ritchie J., Razee H., Shafiee S. (2016). How natural is the supernatural? Synthesis of the qualitative literature from low and middle income countries on cultural practices and traditional beliefs influencing the perinatal period. Midwifery.

[bib0040] Sacks E., Moss W.J., Winch P.J., Thuma P., van Dijk J.H., Mullany L.C. (2015). Skin, thermal and umbilical cord care practices for neonates in southern, rural Zambia: a qualitative study. BMC Pregnancy Childbirth.

[bib0041] Sacks E., Masvawure T.B., Atuyambe L.M., Neema S., Macwan'gi M., Simbaya J., Kruk M. (2017). Postnatal care experiences and barriers to care utilization for home-and facility-delivered newborns in Uganda and Zambia. Matern. Child Health J..

[bib0042] Saving Mothers, Giving Life (2018). 2018 Final report: results of a five-year partnership to reduce maternal and newborn mortality. Retrieved from:http://www.savingmothersgivinglife.org/docs/smgl-final-report.pdf

[bib0043] Scott N.A., Kaiser J.L., Vian T., Bonawitz R., Fong R.M., Ngoma T., Rockers P.C. (2018). Impact of maternity waiting homes on facility delivery among remote households in Zambia: protocol for a quasiexperimental, mixed-methods study. BMJ Open.

[bib0044] Scott N.A., Vian T., Kaiser J.L., Ngoma T., Mataka K., Henry E.G., Hamer D.H. (2018). Listening to the community: Using formative research to strengthen maternity waiting homes in Zambia. PLoS One.

[bib0045] Sialubanje C., Massar K., Hamer D.H., Ruiter R.A. (2015). Reasons for home delivery and use of traditional birth attendants in rural Zambia: a qualitative study. BMC Pregnancy Childbirth.

[bib0046] Sialubanje C., Massar K., Kirch E.M., van der Pijl M.S., Hamer D.H., Ruiter R.A. (2016). Husbands’ experiences and perceptions regarding the use of maternity waiting homes in rural Zambia. Int. J. Gynecol. Obstet..

[bib0047] Sinyange N., Sitali L., Jacobs C., Musonda P., Michelo C. (2016). Factors associated with late antenatal care booking: population based observations from the 2007 Zambia demographic and health survey. Pan Afr. Med. J..

[bib0049] Siwila L.C. (2015). The role of indigenous knowledge in African women's theology of understanding motherhood and maternal health’. Alter. Spec. Edition.

[bib0054] UNICEF, 2016. UNICEF Data: monitoring the situation of children and women. Retrieved fromhttps://data.unicef.org/topic/child-survival/under-five-mortality/#.

[bib0055] UNICEF, 2018. UNICEF Zambia maternal, newborn, and child health. Retrieved fromhttps://data.unicef.org/country/zmb/.

[bib0056] WHO, 1996. Maternity waiting homes: a review of experiences. World Health Organization.

[bib0057] WHO, 2013. WHO traditional medicine strategy 2014–2023. Retrieved fromhttp://apps.who.int/iris/bitstream/handle/10665/92455/9789241506090_eng.pdf?sequence=1.

[bib0061] WHO, 2018b. Complementary feeding. Retrieved fromhttp://www.who.int/nutrition/topics/complementary_feeding/en/.

[bib0062] WHO & UNICEF. (2015). Pregnancy, childbirth, postpartum and newborn care: a guide for essential practice. Retrieved from:https://www.who.int/maternal_child_adolescent/documents/imca-essential-practice-guide/en/. 26561684

